# Combinational Biomarkers for Atrial Fibrillation Derived from Atrial Appendage and Plasma Metabolomics Analysis

**DOI:** 10.1038/s41598-018-34930-6

**Published:** 2018-11-16

**Authors:** Songqing Lai, Xiumeng Hua, Ran Gao, Liang Zeng, Jiangping Song, Jichun Liu, Jing Zhang

**Affiliations:** 1 Cardiothoracic Surgery Department, The First Affiliated Hospital of Nanchang University, Nanchang, 330006 China; 2 State Key Laboratory of Cardiovascular Disease, Fuwai Hospital, National Center for Cardiovascular Diseases, Chinese Academy of Medical Sciences and Peking Union Medical College, Beijing, 100037 China; 3 Yunnan Cancer Hospital, The Third Affiliated Hospital of Kunming Medical University, Yunnan, 650118 China; 4 Cardiovascular Surgery Department, Fuwai Hospital, Chinese Academy of Medical Sciences and Peking Union Medical College, Beijing, 100037 China

## Abstract

Atrial fibrillation (AF) is one of the most common types of arrhythmias and often leads to clinical complications. The objectives of this study were to offer insights into the metabolites of AF and to determine biomarkers for AF diagnosis or prediction. Sixty atrial appendage samples (AF group: 30; non-AF group: 30) and 163 plasma samples (AF group: 48; non-AF group: 115) from 49 AF patients and 116 non-AF patients were subjected to liquid chromatography positive ion electrospray ionization tandem mass spectrometry (LC-ESI-MS/MS) metabolomics analysis. Consequently, 24 metabolites in atrial appendage samples and 24 metabolites in plasma samples were found to reflect metabolic differences between AF and non-AF patients (variable importance in projection (VIP) ≥ 1, P ≤ 0.05). Five identical metabolites including creatinine, D-glutamic acid, choline, hypoxanthine, and niacinamide (VIP ≥ 1.5, P < 0.01, FDR < 0.05) in atrial appendage and plasma samples were considered prominent features of AF patients, and the D-glutamine and D-glutamate metabolic pathway was also identified as a feature of AF patients. Finally, in plasma samples, the combination of D-glutamic acid, creatinine, and choline had an AUC value of 0.927 (95% CI: 0.875–0.979, P < 0.001) and displayed 90.5% sensitivity and 83.3% specificity; this group of metabolites was thus defined as a combinational biomarker for the recognition of AF and non-AF patients.

## Introduction

Atrial fibrillation (AF) is the most common type of arrhythmia and has a substantial effect on individual morbidity and mortality as well as on healthcare expenditure. AF has a prevalence of 0.7% in individuals between the ages of 55 and 59 years; the incidence rises to 17.8% in individuals aged 85 years and over^1,2^. In recent decades, studies have indicated that control of traditional risk factors for cardiovascular disease may not reduce AF to an appreciable extent because there are some other well-established specific risk factors for AF^3^. These factors include age; arterial hypertension; congestive heart failure, including heart failure with impaired or preserved left ventricular systolic function; myocardial infarction^4^; valvular heart disease (VHD); and diabetes mellitus^5^. There are also emerging risk factors for AF, such as subclinical hyperthyroidism, obesity, chronic kidney disease, obstructive sleep apnea, heavy alcohol use, and even high-level endurance training, but the evidence does not clearly indicate that eliminating one or more of these risk factors protects against AF recurrence^6,7^.

Metabolomics is routinely applied as a tool for biomarker discovery to profile metabolites of biofluids, cells, and tissues^8^. Untargeted (global) and targeted mass spectrometry-based metabolomics are the main methodologies for metabolite recovery and identification^9^. These methodologies are based on gas chromatography or liquid chromatography mass spectrometry (GC-MS or LC-MS), which reveals concomitant changes in metabolic pathways including glycolysis, fatty acid β-oxidation, and lipid biosynthesis^8^.

Biomarkers associated with the recurrence and prognosis^10–12^ or the initiation and maintenance^13^ of AF have previously been researched, including inflammatory factors^14^, prothrombotic markers^15^, and microRNAs^16,17^. A nuclear magnetic resonance (NMR) metabolomics technique was used to analyze atrial profibrillatory remodeling in a ventricular-tachypacing (VTP)-induced congestive heart failure (CHF) model in dogs^1^ and in atrial appendage tissues in AF patients^18^, but the results have not been replicated. The NMR technique has its own disadvantages, including low analytical resolution and insensitivity in comparison to mass spectrometry, although it is very powerful in metabolite qualification^19,20^. LC-MS has been deemed to be a fast, high-resolution separation technique similar to ultra-high-performance liquid chromatography (UHPLC), and LC-MS enables the detection of thermolabile molecules^19^. Since 2008, LC-MS has been popular in cardiovascular disease metabolomics analysis^21^. In this study, untargeted LC-MS was utilized to investigate the different metabolites in atrial appendage and plasma samples. To distinguish altered metabolites in the samples and to discover metabolic biomarkers for AF diagnosis or prediction, comparative investigations were performed between AF patients and non-AF patients.

## Results

### Characteristics of the participants

In this study, a total of 165 cardiovascular disease patients were enrolled, including 49 AF patients (AF group) and 116 non-AF patients (non-AF group). There were fewer male patients in the AF group than in the non-AF group (27 (55.10%) vs. 89 (76.72%), P < 0.001). The heart rate (HR) of AF group was higher than that of non-AF group (80.04 ± 11.87 vs. 73.24 ± 10.08, P < 0.001), and the left atrial diameter (LAD) was also larger in the AF group than in the non-AF group (51.83 ± 13.33 mm vs. 38.77 ± 7.82 mm, P < 0.001), but the systolic pressure was lower in the AF group than in the non-AF group (118.85 ± 15.67 vs. 124.28 ± 16.94, P = 0.042). Furthermore, the creatinine clearance rate (Ccr) of the AF group was lower than that of the non-AF group (81.28 ± 21.34 ml/min vs. 94.24 ± 38.85 ml/min, P = 0.023). In addition, the heart failure history of AF group was higher than that of non-AF group (19 (38.78) vs. 23 (19.83), P = 0.011), and the major adverse cardiac and cerebrovascular event (MACCE) incidence were higher in the AF group than in the non-AF group (11 (22.45) vs. 9 (7.76), P = 0.008, respectively) either. Moreover, the patients were split into distinctly different groups based on the New York Heart Association (NYHA) classes of heart failure (I (2, 4.08%), II (12, 24.50%), III (32, 65.30%), and IV (3, 6.12%) for the AF group vs. I (3, 2.59%), II (63, 54.31%), III (49, 42.24%), and IV (1, 0.86%) for the non-AF group, P < 0.001). The other clinical characteristics (Table 1) of the two groups were similar, such as age, BMI, diastolic pressure, ejection fraction (EF), and left ventricular end diastolic diameter (LVEDD).

**Table 1:** Clinical characteristics of the participants (means ± S.D.).

Characteristic	AF group (n = 49)	non-AF group (n = 116)	P value
Age (years)	55.56 ± 9.31	54.31 ± 13.01	0.619
Male, n (%)	27 (55.10)	89 (76.72)	0.000
BMI (kg/m^2^)	24.17 ± 3.69	24.55 ± 3.77	0.550
HR (beats/min)	80.04 ± 11.87	73.24 ± 10.08	0.000
Diastolic pressure (mmHg)	73.65 ± 17.28	71.32 ± 11.60	0.620
Systolic pressure (mmHg)	118.85 ± 15.67	124.28 ± 16.94	0.042
EF%	61.93 ± 10.27	59.51 ± 10.53	0.140
LAD (mm)	51.83 ± 13.33	38.77 ± 7.82	0.000
LVEDD (mm)	51.50 ± 10.73	54.70 ± 10.96	0.066
Creatinine (μmol/L)	80.25 ± 20.56	79.44 ± 19.70	0.720
Ccr (ml/min)	81.28 ± 21.34	94.24 ± 38.85	0.023
Hypertension, n (%)	15 (30.61)	43 (37.07)	0.427
Heart failure history, n (%)	19 (38.78)	23 (19.83)	0.011
COPD, n (%)	2 (4.08)	2 (1.72)	0.730
Diabetes, n (%)	7 (14.29)	21 (18.10)	0.551
MACCE, n (%)	11 (22.45)	9 (7.76)	0.008
NYHA classes, n (%)			0.000
NYHA I	2 (4.08)	3 (2.59)	
NYHA II	12 (24.5)	63 (54.31)	
NYHA III	32 (65.3)	49 (49.24)	
NYHA IV	3 (6.12)	1 (0.86)	

BMI: body mass index; HR: heart rate; EF: ejection fraction; LAD: left atrial diameter; LVEDD: left ventricular end diastolic diameter; Ccr: creatinine clearance rate; COPD: chronic obstructive pulmonary disease; MACCE: major adverse cardiac and cerebrovascular events; NYHA: New York Heart Association.

### Differential metabolite screening

**Figure 1:**
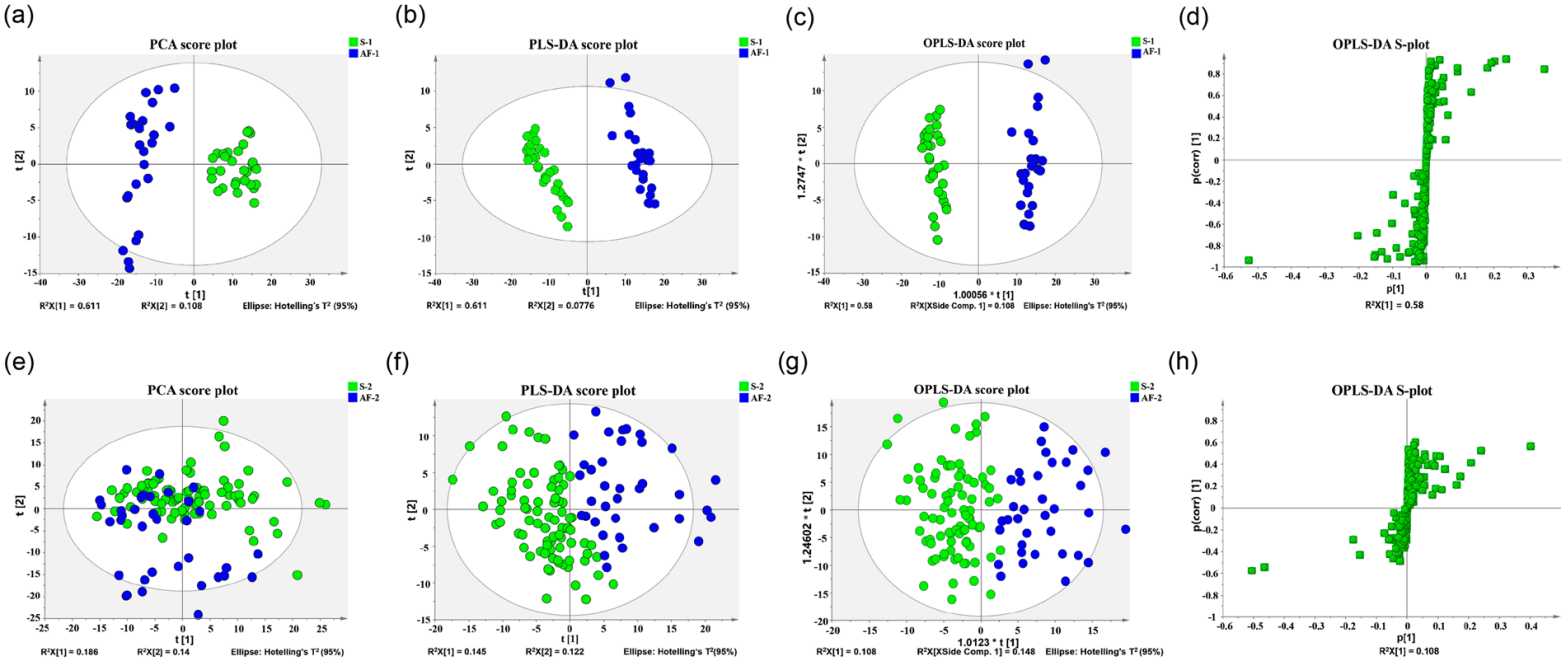
Chemometric analysis of metabolites. (**a**,**e**) PCA score plot of the atrial appendage samples and plasma samples. (**b**,**f**) PLS-DA score plot of LC-MS data for the atrial appendage samples and plasma samples, respectively. (**c**,**g**) OPLS-DA score plot of the atrial appendage sample and plasma sample LC-MS data (S-1 and S-2 denote the atrial appendage and plasma samples of the non-AF group; AF-1 and AF-2 denote the atrial appendage and plasma samples of the AF group). The x-axis represents the samples’ score on the first principal component, and the y-axis represents the samples’ score on the second principal component. R^2^X [1]: the explained variation of the first principal component of the model; R^2^X [2]: the explained variation of the second principal component of the model. (**d**,**h**) OPLS-DA S-plot of LC-MS data for atrial appendage samples and plasma samples, respectively. Each spot represents one compound. The compounds that are near the lower left corner and the upper right corner make greater contributions to the classification of each group.

More than 80% of the metabolites in the LC-MS raw data with null data were deleted. The rest of the null data were replaced with 1/2 of the minimum signal. The acquired LC-MS raw data were normalized. A PCA model helped us delete a few outlier samples; the PCA scores were plotted to determine the aggregation and dispersion of samples (Fig. 1a,e). A PLS-DA score plot helped us to display the classification effect intuitively (Fig. 1b,f). An OPLS-DA score plot helped us to evaluate the classification effect of the PLS model; the classification effect was more significant for the two groups when the degree of separation became larger (Fig. 1c,g). The PCA, PLS-DA, and OPLS-DA scores for the atrial appendage and plasma samples were mainly located within the 95% confidence intervals (Hotelling’s T^2^ ellipse). Validation of the models for the atrial appendage samples was performed. The S-plot of OPLS-DA is shown in Fig. 1d,h. After deleting outliers, there were 55 atrial appendage samples (25 from AF patients and 30 from non-AF patients) and 120 plasma samples (36 from AF patients and 84 from non-AF patients) in our metabolomics analysis study. After mathematical model analysis, 262 and 361 metabolites for the atrial appendage samples and plasma samples were acquired, respectively. Initially, the screening criteria were set as a P value ≤ 0.05 and a variable importance in projection (VIP) value ≥ 1; 24 metabolites for the atrial appendage samples and 24 metabolites for the plasma samples were determined to be potential biomarkers (Supplementary Tables S1 and S2).

Among the 24 metabolites for the atrial appendage samples, 8 metabolites (hypoxanthine, carnitine, 5-aminopentanoic acid, betaine, L-valine, creatinine, choline, and D-glutamic acid) had VIP values above 3.0, which indicated a strong difference between the AF group and the non-AF group. Eleven metabolites (5-aminopentanoic acid, adenosine, betaine, carnitine, creatinine, deoxyguanosine, D-glutamic acid, glycerophosphocholine, L-valine, n-pentadecylamine, and taurine) were significantly increased in the AF group relative to the levels in the non-AF group, whereas the 13 remaining metabolites (butyrylcarnitine, choline, dimethylglycine, guanosine, hypoxanthine, L-acetylcarnitine, L-alanine/sarcosine, L-alpha-aminobutyric acid, L-arginine, L-histidine, L-proline, niacinamide, and pantothenic acid) were all decreased (Supplementary Table S1).

Among the 24 metabolites of the plasma samples, 9 metabolites (choline, niacinamide, betaine, L-valine, L-lactic acid/TF-methoxyacetic acid/glyceraldehyde, creatine, glycerophosphocholine, creatinine, and 3-methyl-2-oxovaleric acid/2-ketohexanoic acid) had VIP values above 3.0, which indicated a strong difference between the AF and non-AF groups. Seven metabolites (L-leucine, glutarate semialdehyde/alpha-ketoisovaleric acid, L-lactic acid/TF-methoxyacetic acid/glyceraldehyde, creatinine, 3-methyl-2-oxovaleric acid/2-ketohexanoic acid, a methacholine-like metabolite, and tagatose/glucose/fructose/galactose/mannose/sorbose/allose) were significantly decreased in the AF group relative to the levels in the non-AF group, whereas the 17 remaining metabolites (choline, oxidized glutathione, niacinamide, betaine, L-valine, D-glutamic acid, gluconic acid, hypoxanthine, creatine, citrulline, glycerophosphocholine, malic acid, (S)-2-methylmalate/(R)-2-methylmalate/2-hydroxyglutarate, acetylcholine/deoxycarnitine, N6,N6,N6-trimethyl-L-lysine, ergothioneine, and N-acetyl-DL-serine) were all increased (Supplementary Table S2).

There were 8 identical metabolites in the atrial appendage samples and the plasma samples of all the patients in the LC-MS metabolomics analysis: betaine, choline, creatinine, D-glutamic acid, glycerophosphocholine, hypoxanthine, L-valine, and niacinamide. Interestingly, relative to those in non-AF patients, 4 identical metabolites in AF patients (betaine, D-glutamic acid, glycerophosphocholine, and L-valine) had the same tendency to increase in all samples, 1 metabolite in AF patients (creatinine) increased in atrial appendage samples but decreased in plasma samples, and 3 metabolites in AF patients (choline, hypoxanthine, and niacinamide) declined in atrial appendage samples but rose in plasma samples. To determine which metabolites caused segregation, a VIP value ≥ 1.5, a P value < 0.01, and a false discovery rate (FDR) < 0.05 were used^22^; based on these criteria, 5 identical metabolites were filtered, including creatinine, D-glutamic acid, choline, hypoxanthine, and niacinamide.

### HCA of differential metabolites and pathways

**Figure 2:**
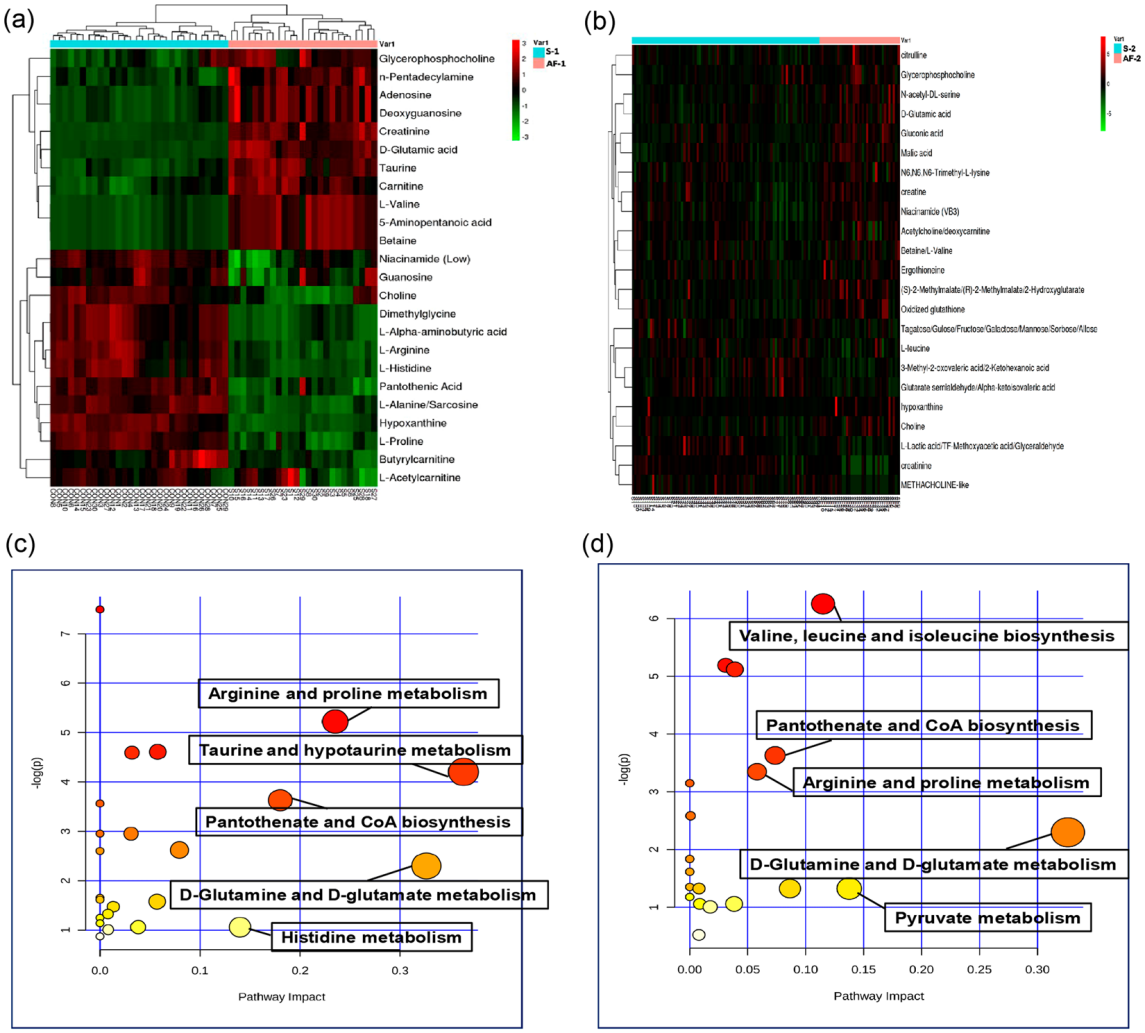
Heatmaps from hierarchical clustering analysis and a diagram of the metabolic pathway enrichment analysis. (**a**) Heatmaps comparing the atrial appendage samples of the non-AF group (S-1) to those of the AF group (AF-1). (**b**) Heatmaps comparing the plasma samples of the non-AF group (S-2) to those of the AF group (AF-2). The rows represent the samples, and the lines represent the metabolites to be identified. The color scale (right) indicates the relative expression levels of the metabolites across all samples; green represents an expression less than the mean, while red represents an expression level greater than the mean. (**c**,**d**) The MetPA analysis based on the KEGG analysis of atrial appendage samples and plasma. Larger circles represent a greater impact of a pathway.

Heatmaps were created through HCA to provide intuitive visualizations of the differences in the patient metabolites between the AF and non-AF groups; comparisons of the atrial appendage samples and the plasma samples of the non-AF group and the AF group are shown in Fig. 2a,b, respectively. The heatmaps clearly show that the metabolic profiles of the AF group were more easily distinguished from those of the non-AF group in the atrial appendage samples than in the plasma samples.

KEGG analysis of the differential metabolites in the atrial appendage samples and plasma samples are shown in Tables 2 and 3. There were 7 significantly altered pathways (P < 0.05) involving atrial appendage sample metabolism (Table 2): ‘aminoacyl-tRNA biosynthesis’, ‘arginine and proline metabolism’, ‘glycine, serine, and threonine metabolism’, ‘purine metabolism’, ‘taurine and hypotaurine metabolism’, ‘pantothenate and CoA biosynthesis’, and ‘beta-alanine metabolism’, all of which showed ≥ 2 hits. Similarly, there were 6 significantly altered pathways (P < 0.05) involving plasma sample metabolism (Table 3): ‘valine, leucine, and isoleucine biosynthesis’, ‘glycerophospholipid metabolism’, ‘valine, leucine, and isoleucine degradation’, ‘pantothenate and CoA biosynthesis’, ‘arginine and proline metabolism’ and ‘propanoate metabolism’, all of which also had ≥ 2 hits. Next, MetPA analysis of the differential metabolites was performed (Fig. 2c,d).

**Table 2:** KEGG analysis of the differential metabolites in atrial appendage samples.

Pathway	Total	Expected	Hits	Raw P	-log(P)	Holm adjust	FDR	Impact
Aminoacyl-tRNA biosynthesis	75	0.72	5	0.00	7.49	0.04	0.04	0.00
Arginine and proline metabolism	77	0.74	4	0.01	5.22	0.43	0.20	0.24
Glycine, serine and threonine metabolism	48	0.46	3	0.01	4.61	0.78	0.20	0.06
Purine metabolism	92	0.88	4	0.01	4.59	0.78	0.20	0.03
Taurine and hypotaurine metabolism	20	0.19	2	0.01	4.20	1.00	0.24	0.36
Pantothenate and CoA biosynthesis	27	0.26	2	0.03	3.63	1.00	0.32	0.18
beta-Alanine metabolism	28	0.27	2	0.03	3.56	1.00	0.32	0.00
Nitrogen metabolism	39	0.37	2	0.05	2.95	1.00	0.46	0.00
Glycerophospholipid metabolism	39	0.37	2	0.05	2.95	1.00	0.46	0.03
Lysine degradation	47	0.45	2	0.07	2.62	1.00	0.54	0.08
D-Arginine and D-ornithine metabolism	8	0.08	1	0.07	2.60	1.00	0.54	0.00
D-Glutamine and D-glutamate metabolism	11	0.11	1	0.10	2.30	1.00	0.67	0.33
Selenoamino acid metabolism	22	0.21	1	0.19	1.65	1.00	1.00	0.00
Ether lipid metabolism	23	0.22	1	0.20	1.61	1.00	1.00	0.00
Alanine, aspartate and glutamate metabolism	24	0.23	1	0.21	1.58	1.00	1.00	0.06
Valine, leucine and isoleucine biosynthesis	27	0.26	1	0.23	1.47	1.00	1.00	0.01
Vitamin B6 metabolism	32	0.31	1	0.27	1.32	1.00	1.00	0.01
Propanoate metabolism	35	0.33	1	0.29	1.25	1.00	1.00	0.00
Valine, leucine and isoleucine degradation	40	0.38	1	0.32	1.14	1.00	1.00	0.00
Nicotinate and nicotinamide metabolism	44	0.42	1	0.35	1.06	1.00	1.00	0.04
Histidine metabolism	44	0.42	1	0.35	1.06	1.00	1.00	0.14
Primary bile acid biosynthesis	47	0.45	1	0.37	1.01	1.00	1.00	0.01
Cysteine and methionine metabolism	56	0.54	1	0.42	0.87	1.00	1.00	0.00

Total: the number of metabolites in each metabolic pathway. Hits: the number of differential metabolites in the target metabolic pathway. Raw P: P value of the hypergeometric test. Holm adjust: P value after the Holm false positive adjustment. FDR: false discovery rate. Impact: metabolic pathway influence value (a higher value denotes a stronger influence).

**Table 3:** KEGG analysis of the differential metabolites in plasma samples.

Pathway	Total	Expected	Hits	Raw P	-log(P)	Holm adjust	FDR	Impact
Valine, leucine and isoleucine biosynthesis	27	0.26	3	0.00	6.26	0.15	0.15	0.12
Glycerophospholipid metabolism	39	0.37	3	0.01	5.19	0.44	0.16	0.03
Valine, leucine and isoleucine degradation	40	0.38	3	0.01	5.12	0.47	0.16	0.04
Pantothenate and CoA biosynthesis	27	0.26	2	0.03	3.63	1	0.53	0.07
Arginine and proline metabolism	77	0.74	3	0.04	3.35	1	0.56	0.06
Propanoate metabolism	35	0.33	2	0.04	3.15	1	0.57	0.00
Glycine, serine and threonine metabolism	48	0.46	2	0.08	2.58	1	0.86	0.00
D-Glutamine and D-glutamate metabolism	11	0.11	1	0.10	2.30	1	1	0.33
Aminoacyl-tRNA biosynthesis	75	0.72	2	0.16	1.84	1	1	0.00
Ether lipid metabolism	23	0.22	1	0.20	1.61	1	1	0.00
Glycolysis or gluconeogenesis	31	0.30	1	0.26	1.35	1	1	0.00
Vitamin B6 metabolism	32	0.31	1	0.27	1.32	1	1	0.01
Pentose phosphate pathway	32	0.31	1	0.27	1.32	1	1	0.09
Pyruvate metabolism	32	0.31	1	0.27	1.32	1	1	0.14
Glutathione metabolism	38	0.36	1	0.31	1.18	1	1	0.00
Histidine metabolism	44	0.42	1	0.35	1.06	1	1	0.01
Nicotinate and nicotinamide metabolism	44	0.42	1	0.35	1.06	1	1	0.04
Lysine degradation	47	0.45	1	0.37	1.01	1	1	0.02
Purine metabolism	92	0.88	1	0.59	0.52	1	1	0.01

Total: the number of metabolites in each metabolic pathway. Hits: the number of differential metabolites in the target metabolic pathway. Raw P: P value of the hypergeometric test. Holm adjust: P value after the Holm false positive adjustment. FDR: false discovery rate. Impact: metabolic pathway influence value (a higher value denotes a stronger influence).

An impact value greater than 0.10 indicates that the altered pathways clearly affect AF^23^, so we filtered out 5 metabolic pathways (‘taurine and hypotaurine metabolism’, ‘D-glutamine and D-glutamate metabolism’, ‘arginine and proline metabolism’, ‘pantothenate and CoA biosynthesis’, and ‘histidine metabolism’) in the atrial appendage samples and 3 metabolic pathways (‘valine, leucine and isoleucine biosynthesis’, ‘pyruvate metabolism’, and ‘D-glutamine and D-glutamate metabolism’) in the plasma samples. After comparing the two groups, we determined the ‘D-glutamine and D-glutamate metabolism’ pathway to be the main canonical pathway in AF, which is consistent with the fact that D-glutamic acid is a prominent feature of AF in cardiovascular disease patients^24^.

### Differential metabolite correlation heatmap

**Figure 3:**
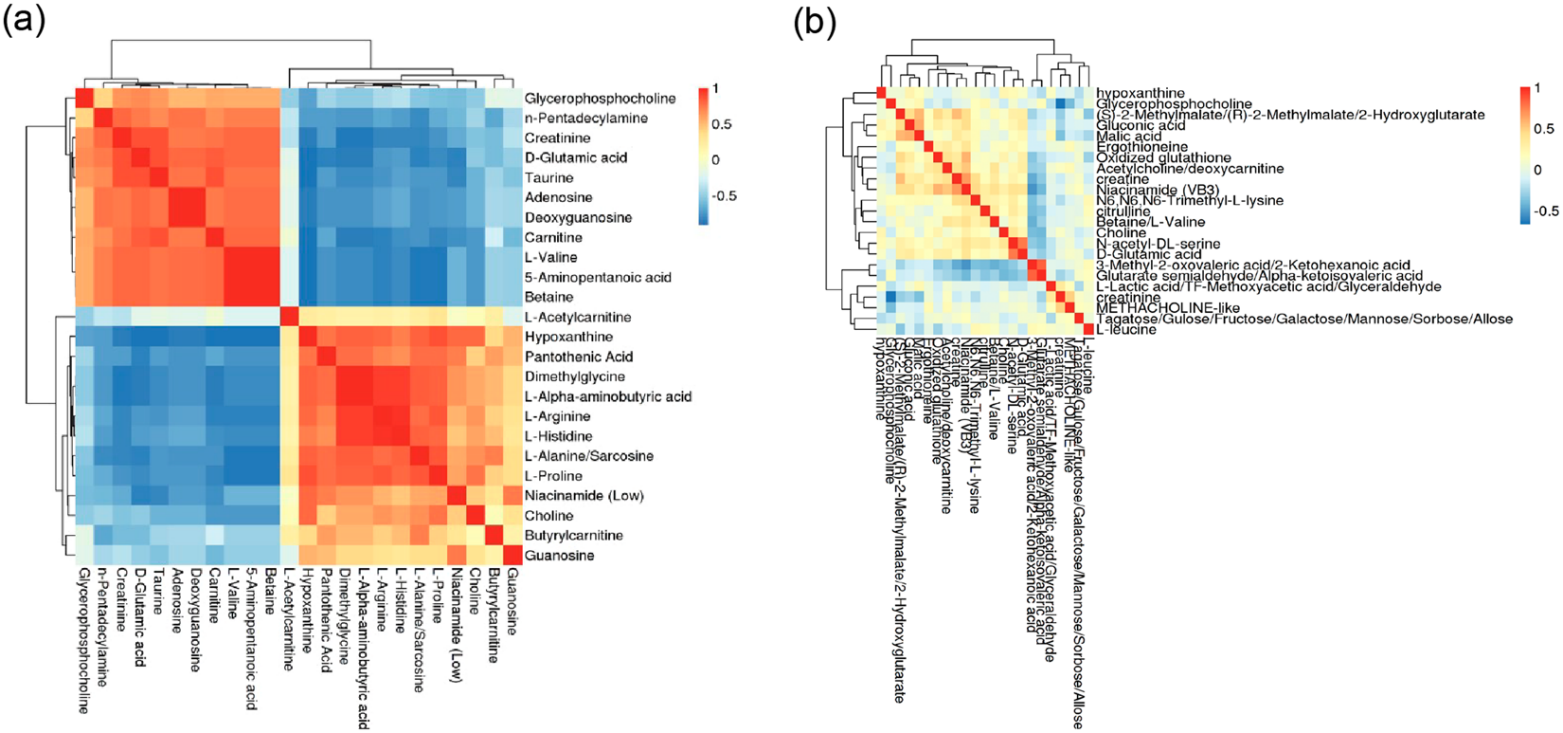
Correlation heatmaps of differential metabolites. (**a**) Differential metabolite correlation heatmaps for atrial appendage samples. (**b**) Differential metabolite correlation heatmaps for plasma samples. The color scale (right) indicates the degree of correlation of the differential metabolites; red represents a positive correlation, while blue represents a negative correlation. In addition, 1 and −1 indicate the strongest positive and negative correlations, respectively.

Differential metabolite correlation heatmaps for the atrial appendage samples and the plasma samples are shown in Fig. 3. Among the atrial appendage metabolites, the first 11 metabolites (from glycerophosphocholine to betaine) positively correlated with each other but negatively correlated with the last 13 metabolites (from L-acetylcarnitine to guanosine). Likewise, the last 13 metabolites were positively correlated with each other. Interestingly, the degrees of correlation of the positive and negative correlations were relatively strong, as the colors were much darker (Fig. 3a).

Among the plasma metabolites, the 16 increased metabolites (from hypoxanthine to D-glutamic acid on the right side) were nearly positively correlated with each other but were negatively correlated to the last 7 metabolites (from 3-methyl-2-oxovaleric acid/2-ketohexanoic acid to L-leucine), which were decreased and were positively correlated with each other. The metabolites 3-methyl-2-oxovaleric acid/2-ketohexanoic acid and glutarate semialdehyde/alpha-ketoisovaleric acid as well as D-glutamic acid and N-acetyl-DL-serine showed positive degrees of correlation reaching 0.8. However, glycerophosphocholine was negatively correlated (degree of correlation: −1.0) with creatinine (Fig. 3b).

### Statistical analysis for the identified differential metabolites

After the differential metabolite screening, Z-scores were used for statistical analysis (see Supplementary Fig. S1). The Z-scores were transformed based on the relative content of the metabolites. It was found that the degree of variation of the metabolites in the different groups was considerable in this study. Relative to Z-score of those in the non-AF group (S-1, S-2), the Z-score of the atrial appendage samples in the AF group was in the range of −2 to 3 (Fig. S1a), and the Z-score of the plasma samples in the AF group was in the range of −3 to 4 (Fig. S1b). Although the Z-score range of the atrial appendage samples was smaller, the plots were more diffuse, and the difference between the AF and non-AF groups was more obvious, suggesting that the levels of metabolites in the atrial appendage samples varied more widely than those in the plasma samples.

### ROC curve analysis of the differential metabolites

**Figure 4:**
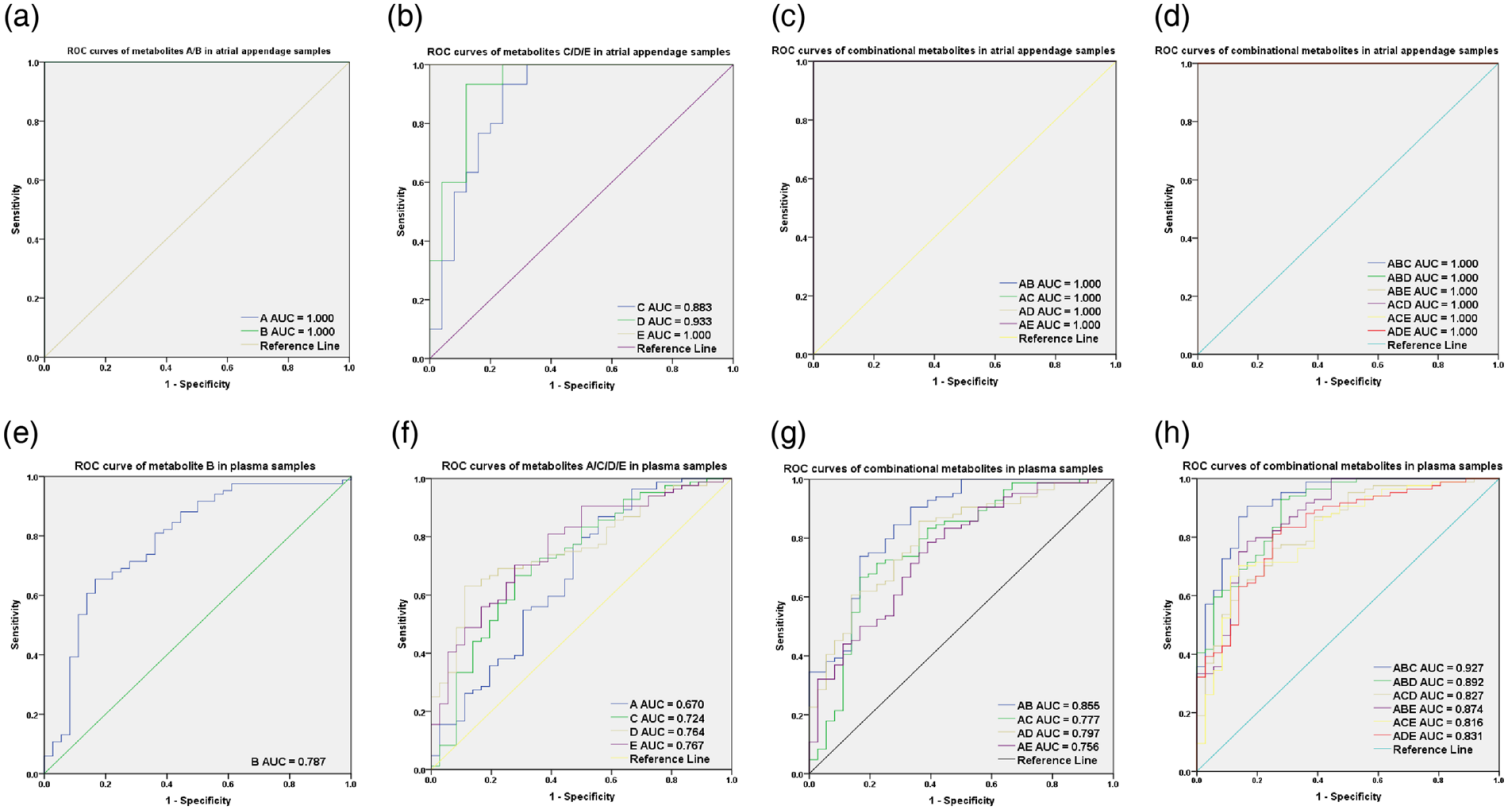
ROC curve analysis of differential metabolites. (**a–d**) ROC curve analysis of the atrial appendage samples. (**e–g**) ROC curve analysis of the plasma samples. The metabolites A, B, C, D, and E represent D-glutamic acid, creatinine, choline, niacinamide, and hypoxanthine, respectively. The sensitivity (true positive rate) was set as the ordinate, and 1-specificity (false positive rate) was set as the abscissa. An AUC value approaching 1.0 indicates a better diagnostic effectiveness.

After the screening and statistical analysis described above, 5 identical metabolites (creatinine, D-glutamic acid, choline, hypoxanthine, and niacinamide) and one cardinal pathway (‘D-glutamine and D-glutamate metabolism’) had been filtered, indicating that the metabolite D-glutamic acid is critical in the pathophysiological mechanisms of AF. Thus, we drew ROC curves based on D-glutamic acid. The ROC curves are shown in Fig. 4. The metabolites A, B, C, D, and E represent D-glutamic acid, creatinine, choline, niacinamide, and hypoxanthine, respectively. The optimal cutoff values were chosen to calculate the sensitivity (true positive rate, TPR) and specificity (true negative rate, TNR) for the ROC curves (Supplementary Table S3).

Among the metabolites of the atrial appendage samples, metabolite C (AUC = 0.883, 95% CI: 0.785–0.980, P < 0.001) showed a high diagnostic effectiveness (Fig. 4b, Supplementary Table S3). Metabolite D (AUC = 0.933, 95% CI: 0.864–1.000, P < 0.001) and metabolites A/B/E (AUC = 1.000, 95% CI: 1.000–1.000, P < 0.001), as well as their combinational markers, exhibited excellent diagnostic effectiveness in distinguishing AF from non-AF in cardiovascular disease patients (Fig. 4a–d, Supplementary Table S3).

Likewise, among the metabolites of the plasma samples, metabolite A had AUC values less than 0.7, metabolites B/C/D/E and the combinational markers AC/AD/AE had higher AUC values between 0.7 and 0.8 (P < 0.001), and metabolites AB/ABD/ABE/ACD/ACE/ADE had AUC values greater than 0.8 but less than 0.9 (P < 0.001) (Fig. 4e–h, Supplementary Table S3). However, none of the metabolites had a sensitivity greater than 0.9 and a specificity greater than 0.8 except the combinational marker ABC (Supplementary Table S3). The combinational marker ABC, which comprises D-glutamic acid, creatinine, and choline, displayed an AUC value of 0.927 (95% CI: 0.875–0.979, P < 0.001), while its diagnostic effectiveness reached 90.5% sensitivity and 83.3% specificity for the prediction of AF in plasma samples (Fig. 4h, Supplementary Table S3).

## Discussion

After decades of research and therapeutic intervention for AF, scientists are still striving to understand the causes and mechanisms of the condition. Biomarkers derived from blood, such as markers of coagulation, renal function, inflammation, myocardial injury, and cardiovascular stress, have been associated with clinical events^3^. Biomarkers derived from the blood may reflect the current disease state or the prognosis of diseases. Accompanied by the development and progression of AF, metabolite biomarkers might reflect the pathophysiological mechanisms of AF. Mayr *et al*.^18^ performed an untargeted NMR-based metabolomic analysis to demonstrate that both β-hydroxybutyrate and its ketogenic amino acid as well as glycine were increased in AF patients’ atrial myocardial samples. This NMR-based metabolomic analysis focused on postoperative AF. However, the NMR technique has its own disadvantages, as previously mentioned. Furthermore, an ideal innovative biomarker for diagnosis or prognosis should be able to be measured through minimally invasive techniques, should have high sensitivity and specificity, and should have good operability and repeatability to be feasible for the patients. Therefore, biomarkers in the blood are worth continued consideration.

In this study, 60 atrial appendage samples and 163 plasma samples from 165 cardiovascular disease patients (49 AF patients and 116 non-AF patients) were subjected to LC-MS/MS-based metabolomics to investigate whether AF alters the metabolism of cardiovascular disease patients. Moreover, untargeted metabolites in tissue and plasma were compared to determine whether the differential metabolites could be potential biomarkers for AF diagnosis or prediction. Twenty-four metabolites from plasma samples and 24 metabolites from atrial appendage samples were screened for their ability to distinguish AF from non-AF patients (Supplementary Tables S1 and S2).

Eight identical metabolites were identified between the atrial appendage samples and the plasma samples before the comparison of expression in this study (Supplementary Tables S1 and S2). Betaine^1^, choline^1,18^, and L-valine^18^ have been reported in the literature to be associated with AF, and our LC-MS/MS metabolites added to this consensus. Betaine has been reported to be a novel biomarker for colorectal cancer^25^. Choline can be oxidized to betaine but shows the opposite relationship in metabolic syndrome as a key component correlated with mitochondrial dysfunction^26^. This finding suggests that the decreased choline and the increased betaine in the atrial appendage samples of AF patients may well be associated with mitochondrial dysfunction. Creatinine has been reported to be significantly decreased in the blood samples of patients with AF with hypertension^27^, and our results also showed that creatinine was decreased in the plasma samples. Furthermore, we found that creatinine increased in the atrial appendage samples of AF patients. The most likely reason is that cardiomyocytes uptake creatinine from the extracellular matrix, leading to AF; however, this possibility needs further investigation. Hypoxanthine has been reported to accumulate substantially in the ischemic pig myocardium^28^; hypoxanthine was increased in the plasma samples but was decreased in the atrial appendage samples in our study. Hypoxanthine is involved in the purine metabolism pathway. Hypoxanthine can be metabolized to xanthine and uric acid by xanthine oxidase^29^. Ordinarily, the endogenous cause of increased hypoxanthine is hypoxanthine-guanine phosphoribosyltransferase (HGPRT) deficiency, which leads to gout, hyperuricemia, and Lesch-Nyhan syndrome^30^. Researchers have demonstrated that hypoxanthine can cause endothelial dysfunction through oxidative stress-induced apoptosis^29^. To a certain extent, our results hint that hypoxanthine imbalance can result in AF. Additionally, glycerophosphocholine has been reported to accumulate in human aortic tissue and plasma in response to early atherosclerosis^18^; in our study, glycerophosphocholine was increased in the atrial appendage samples and plasma samples of AF patients. D-glutamic acid belongs to the group of D-amino acids, which are metabolized only by D-aspartate oxidase (DDO) in mammals^31^ and are now thought to be naturally occurring physiologically active substances and biomarkers^32,33^. D-glutamic acid might activate transporter-associated Cl^−^ conductance and has been reported to regulate neuronal transmission^34^. Kazuaki *et.al*. demonstrated that in 2-DM patients, preserved left ventricular ejection fraction reduced heart rate recovery and was associated with AF^35^, illustrating that autonomic neuropathy seems to be involved in the pathogenesis of AF. Thus, D-glutamic acid may influence the autonomic nervous system, resulting in AF. Furthermore, our results from the LC-MS/MS metabolomics analysis regarding hypoxanthine, D-glutamic acid, and niacinamide suggest that these metabolites might be novel biomarkers associated with AF, but this conclusion should be closely inspected.

We used a VIP value ≥ 1.5, a P value < 0.01, and an FDR < 0.05 to screen the metabolites^22^, and 5 identical metabolites (creatinine, D-glutamic acid, choline, hypoxanthine, and niacinamide) in the samples from the two groups were filtered (Supplementary Table S1 and S2).

KEGG analysis (Tables 2 and 3) and metabolic pathway enrichment analysis (Fig. 2c,d) were performed to identify the potential metabolic pathways perturbed in AF. An impact value greater than 0.10 for the altered pathways has been deemed to reflect a clear influence on AF^23^. Only one common pathway (‘D-glutamine and D-glutamate metabolism’) was selected from the samples of the two groups. The fact that the ‘D-glutamine and D-glutamate metabolism’ pathway had an impact value greater than 0.10 (0.33) suggests that the ‘D-glutamine and D-glutamate metabolism’ pathway and the metabolite D-glutamic acid are prominent features of AF in cardiovascular disease patients. The KEGG analysis offered us a foundation on which to determine that the metabolite D-glutamic was indispensable to AF.

Receiver operating characteristic (ROC) curves have been successfully applied to evaluate the diagnostic accuracy of biomarkers in many studies^22,36^. An area under the curve (AUC) greater than 0.7 represents very high diagnostic effectiveness, and the effectiveness is particularly good when the AUC value is greater than 0.9^37^. ROC curves based on D-glutamic acid were drawn to explicitly determine which metabolites can definitely diagnose AF in patients. Our results revealed that the combinational marker comprised of D-glutamic acid, creatinine, and choline had an AUC value of 0.927 (95% CI: 0.875–0.979, P < 0.001), while the diagnostic effectiveness of this combinational biomarker achieved 90.5% sensitivity and 83.3% specificity, demonstrating that its ability to predict AF in plasma samples was satisfactory (Fig. 4h, supplementary Table S3). In this study, we performed LC-MS metabolomics on atrial appendage samples and plasma samples from patients with or without AF. Our results demonstrate that measuring changes in metabolite patterns is a feasible method for evaluating the clinical characteristics of AF. In addition, we screened an affected pathway, the ‘D-glutamine and D-glutamate metabolism’ pathway. The plasma metabolites creatinine, D-glutamic acid, and choline were defined as a combinational biomarker to recognize AF and non-AF on the basis of the patients’ blood. However, future comparison with the results of a metabolomics analysis with a larger sample size is needed to validate our findings.

## Materials and Methods

### Participants

**Figure 5:**
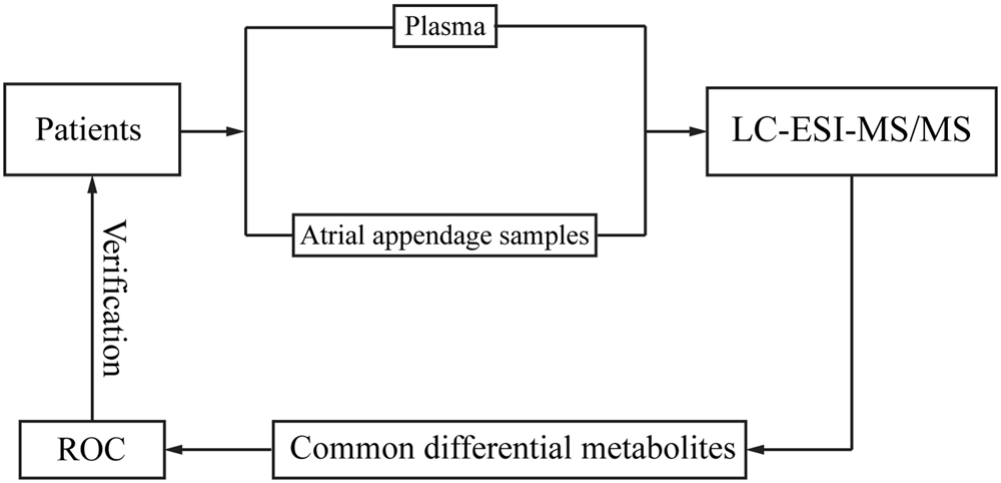
The workflow of the metabolomics analysis of the plasma and atrial appendage samples from AF and non-AF patients.

Related investigations were conducted under the approval of the Ethics Committees of the Fuwai Hospital, National Center for Cardiovascular Diseases, Chinese Academy of Medical Sciences and Peking Union Medical College (Beijing, China). All participants provided written informed consent at the time of enrollment. Two groups of cardiovascular disease patients, an AF group (49 AF patients) and a non-AF group (116 non-AF patients), were included in the present study. Sixty atrial appendage samples and 163 plasma samples were collected from the patients at Fuwai Hospital from May 2015 to March 2016. Atrial appendage samples were obtained from cardiovascular disease patients who had surgical treatment. All methods were performed in accordance with the relevant guidelines and regulations. The datasets and methods, including the methods of data acquisition and analysis, are available from the corresponding author upon reasonable request. The workflow of our study is shown in Fig. 5.

### Sample collection and preparation

Atrial appendage samples were collected during surgery following surgical principles. The atrial appendage was cut into small pieces, transferred to a clean tube, and immediately stored at −80 °C until use. The blood samples were collected in ethylenediaminetetraacetic acid (EDTA)-coated anticoagulation tubes the morning after the patients were hospitalized. The collected blood was immediately centrifuged to isolate the plasma at 3,000 × *g* for 10 min at 4 °C, and the clear supernatant was transferred to a new Eppendorf tube. All plasma samples were promptly frozen in a −80 °C freezer until analysis. Sample preparation followed a method that has been described in recent literature^38^. Frozen samples were thawed at room temperature, extracted in 75% aqueous methanol containing 0.1% formic acid, and analyzed by untargeted LC-MS as follows.

### LC-ESI-MS/MS analysis

Methyl alcohol, acetonitrile, and all other materials and chemicals were of HPLC grade and were obtained from Sigma-Aldrich Co. (USA). Ultrapure water (Milli-Q) was obtained from a Milli-Q Gradient Direct 16 system (Millipore, USA). Sample analysis was performed using an electrospray ionization (ESI) TSQ Vantage triple quadrupole quantum mass spectrometer (Thermo Scientific, USA) with a Surveyor Autosampler combined with an Agilent 1200 Series HPLC System (Agilent Technologies, Waldbronn, Germany) equipped with Xcalibur 3.0 software (Thermo Finnigan, USA).

### Data processing

Raw data acquired from the HPLC-MS systems were converted into databases with equal quality and retention time peak alignment. Then, peak discrimination, total peak area normalization, filtering, alignment, and matching were performed. Afterward, annotated metabolites were put through SIMCA-P 13.0 (Umetrics AB, Umea, Sweden) for statistical analysis. Principal component analysis (PCA) was conducted using the Pareto model as an unsupervised analysis to view the clustering trend while removing outlier samples. Later, partial least squares discriminant analysis (PLS-DA) and orthogonal partial least squares discriminant analysis (OPLS-DA) were employed as a supervised analysis using a permutation test (200 permutations) to prevent overfitting of the PLS-DA model^39^. PLS-DA and OPLS-DA were performed to clarify the groups among clusters^40^, which was particularly helpful for detecting and removing abnormal samples to improve the accuracy of the model. Finally, Student’s t-test (P < 0.05) was combined with the variable importance in projection (VIP) value^41^ of the first principal component or the data were visualized using the S-plot^42^ in OPLS-DA to search for distinct metabolites.

Heatmap analysis was used to further study the relationships between metabolites and samples, where the intent was to discern similar metabolic profiles among samples or identical metabolic activity among metabolites. Heatmaps were created using the pheatmap R package (Kolde R. Pheatmap: Pretty Heatmaps; R package version 1.0.8; https://cran.r-project.org/web/packages/pheatmap/index.html). Cluster analysis or clustering is always used to determine the metabolic patterns of metabolites under different experimental conditions. Hierarchical clustering analysis (HCA) of samples in the heatmap was performed using the average linkage clustering method and Euclidian distances. Metabolites with the same metabolic pattern might possess the same function or participate in the same metabolic process or cellular pathway.

Additionally, metabolite-metabolite correlation analysis (MMCA)^43^ was performed with Pearson’s product-moment correlation (Pearson’s r) in R statistical software. MMCA offers a new insight into metabolomics. For instance, similar trends in variation among metabolites indicate positive correlations, while different trends in variation indicate negative correlations. When the linear correlation of two metabolites is enhanced, the correlation coefficient tends toward 1 or −1 for a positive correlation or a negative correlation, respectively. The correlation matrix was calculated through the cor function in R (v3.1.3) software (https://www.r-project.org/). The metabolite correlation heatmap was drawn based on the correlation coefficients.

Standard scores, also called Z-scores, which are used to identify distinct metabolites, can evaluate the relative contents of the metabolites in each sample. The formula for the Z-score is z = (x − μ)/σ (x, specific score of the metabolites; μ, mean; σ standard deviation).

The Kyoto Encyclopedia of Genes and Genomes (KEGG)^44^ (http://www.genome.jp/kegg/) is a database resource that integrates genomic, chemical, and systemic functional information. KEGG analysis was used to analyze the interrelationships of the metabolites in the metabolic pathways. In addition, enrichment analysis was performed with MetaboAnalyst 3.0 (www.metaboanalyst.ca), a comprehensive server for metabolomic data analysis, including three functional analyses: enrichment analysis, pathway analysis, and integrated pathway analysis^45^. MetPA is a part of MetaboAnalyst that is mainly based on the KEGG metabolic pathways^46^. MetPA uses pathway enrichment analysis and pathway topological analysis to identify the potential metabolic pathways interfered with by biological perturbation. The topological analysis selected in this study was ‘relative betweenness centrality’. Furthermore, MetPA can analyze the related metabolic pathways of different metabolites between two groups through hypergeometric tests.

### Statistical analysis

Statistical analysis was performed with SPSS 22.0 software. Continuous variables were expressed as the means ± S.D., and categorical variables were presented as counts and percentages (%). Continuous variables were calculated by nonparametric tests or t-tests, categorical variables were analyzed using chi-square or Fisher’s tests, and multilevel data analysis was performed with the Mann-Whitney U test. A two-tailed P value < 0.05 was considered statistically significant. ROC curves and AUCs were calculated in SPSS 22.0 software (IBM, USA), but the combinational marker ROC curves first required binary logistic regression.

## Electronic supplementary material

Supplementary Information

## Data Availability

Raw data are provided in the Supplementary Information of this article. Other data are available from the corresponding author (zhangjingfw@163.com).
