# Supplementary material for: Combinational Biomarkers for Atrial Fibrillation Derived from Atrial Appendage and Plasma Metabolomics Analysis
Source: Sci Rep. 2018 Nov 16;8:16930. doi: 10.1038/s41598-018-34930-6 (PMC6240090; doi:10.1038/s41598-018-34930-6)
Supplement: Supplementary file 1 — Supplementary Information [file 41598_2018_34930_MOESM1_ESM.doc]

## Combinational Biomarkers for Atrial Fibrillation Derived from Atrial Appendage and Plasma Metabolomics Analysis

Songqing Lai^1, *^, Xiumeng Hua^2, *^, Ran Gao^3^, Liang Zeng^1^, Jiangping Song^2^, Jichun Liu^1, +^, Jing Zhang^4, +^

^1^ Cardiothoracic Surgery Department, The First Affiliated Hospital of Nanchang University, Nanchang, 330006, China

^2^ State Key Laboratory of Cardiovascular Disease, Fuwai Hospital, National Center for Cardiovascular Diseases, Chinese Academy of Medical Sciences and Peking Union Medical College, Beijing, 100037, China

^3^ Yunnan Cancer Hospital, The Third Affiliated Hospital of Kunming Medical University, Yunnan, 650118, China

^4^ Cardiovascular Surgery Department, Fuwai Hospital, Chinese Academy of Medical Sciences and Peking Union Medical College, Beijing, 100037, China

*&^+^ These authors contributed equally to this work.

Correspondence and requests for materials should be addressed to J.Z.(zhangjingfw@163.com) or J.C.L.(Liujichun999@163.com). Postal address: 167A Beilishi Road, Xi Cheng District, Beijing 100037, China. Tel.: 086-10-88392036. Fax: 086-10-88392036.

**Table S1. Differential metabolites of atrial appendage samples obtained from LC-ESI-MS/MS metabolomics.**

| Compounds | Splot p | splot p(corr) | VIP | FC | log_2_(FC) | P value | FDR | AUC |
| --- | --- | --- | --- | --- | --- | --- | --- | --- |
| 5-aminopentanoic acid | 0.2377 | 0.9407 | 3.5595 | 1.6741 | 0.7433 | 0.0000 | 0.0000 | 0.9973 |
| Adenosine | 0.1817 | 0.8579 | 2.7071 | 4.1632 | 2.0577 | 0.0000 | 0.0000 | 0.9933 |
| Betaine | 0.2377 | 0.9407 | 3.5595 | 1.6741 | 0.7433 | 0.0000 | 0.0000 | 0.9973 |
| Butyrylcarnitine | -0.1012 | -0.5955 | 1.6464 | 0.4520 | -1.1456 | 0.0000 | 0.0000 | 0.9187 |
| Carnitine | 0.3506 | 0.8457 | 5.2554 | 1.3728 | 0.4571 | 0.0000 | 0.0000 | 0.9867 |
| Choline | -0.2026 | -0.7088 | 3.0529 | 0.6365 | -0.6517 | 0.0000 | 0.0000 | 0.8827 |
| Creatinine | 0.2030 | 0.9201 | 3.1277 | 2.1820 | 1.1257 | 0.0000 | 0.0000 | 1.0000 |
| Deoxyguanosine | 0.1817 | 0.8579 | 2.7071 | 4.1632 | 2.0577 | 0.0000 | 0.0000 | 0.9933 |
| D-Glutamic acid | 0.1939 | 0.9038 | 3.0106 | 4.3466 | 2.1199 | 0.0000 | 0.0000 | 1.0000 |
| Dimethylglycine | -0.0780 | -0.9051 | 1.1624 | 0.3983 | -1.3280 | 0.0000 | 0.0000 | 0.9987 |
| Glycerophosphocholine | 0.1337 | 0.6301 | 2.0256 | 1.7468 | 0.8048 | 0.0000 | 0.0000 | 0.8680 |
| Guanosine | -0.0631 | -0.4084 | 1.0132 | 0.7425 | -0.4296 | 0.0006 | 0.0010 | 0.7640 |
| Hypoxanthine | -0.5265 | -0.9403 | 7.9762 | 0.4219 | -1.2452 | 0.0000 | 0.0000 | 1.0000 |
| L-acetylcarnitine | -0.0977 | -0.3278 | 1.2359 | 0.8934 | -0.1626 | 0.0393 | 0.0510 | 0.6627 |
| L-alanine/sarcosine | -0.1152 | -0.9264 | 1.7749 | 0.4424 | -1.1765 | 0.0000 | 0.0000 | 1.0000 |
| L-alpha-aminobutyric acid | -0.0780 | -0.9051 | 1.1624 | 0.3983 | -1.3280 | 0.0000 | 0.0000 | 0.9987 |
| L-arginine | -0.1519 | -0.8854 | 2.2216 | 0.2941 | -1.7658 | 0.0000 | 0.0000 | 0.9947 |
| L-histidine | -0.1327 | -0.8604 | 1.9575 | 0.2876 | -1.7980 | 0.0000 | 0.0000 | 0.9933 |
| L-proline | -0.1534 | -0.9065 | 2.2778 | 0.5474 | -0.8692 | 0.0000 | 0.0000 | 0.9853 |
| L-valine | 0.2377 | 0.9407 | 3.5595 | 1.6741 | 0.7433 | 0.0000 | 0.0000 | 0.9973 |
| Niacinamide (Low) | -0.1462 | -0.6819 | 2.2976 | 0.7581 | -0.3995 | 0.0000 | 0.0000 | 0.9333 |
| n-pentadecylamine | 0.0932 | 0.8200 | 1.4089 | 1.6464 | 0.7193 | 0.0000 | 0.0000 | 0.9987 |
| Pantothenic acid | -0.0880 | -0.8256 | 1.3271 | 0.5690 | -0.8134 | 0.0000 | 0.0000 | 0.9600 |
| Taurine | 0.0935 | 0.8591 | 1.4602 | 2.2555 | 1.1734 | 0.0000 | 0.0000 | 0.9867 |

S-plot p: the magnitude variable of the S-plot X dataset; S-plot p(corr): the reliability value of the S-plot X dataset; VIP: the variable importance in projection score; FC: fold change; log_2_(FC): log_2_ value of fold change; P value: P value of the Wilcoxon signed-rank test; FDR: false discovery rate; AUC: area under the ROC curve.

**Table S2. Differential metabolites of plasma samples obtained from LC-ESI-MS/MS metabolomics.**

| Compounds | Splot p | Splot p(corr) | VIP | FC | log_2_(FC) | P value | FDR | AUC |
| --- | --- | --- | --- | --- | --- | --- | --- | --- |
| Choline | -0.0012 | -0.0982 | 6.0764 | 1.2765 | 0.3522 | 0.0001 | 0.0013 | 0.7239 |
| L-leucine | 0.1613 | 0.2115 | 1.0338 | 0.9012 | -0.1501 | 0.0093 | 0.0378 | 0.6505 |
| Oxidized glutathione | -0.0023 | -0.0856 | 1.1452 | 1.7645 | 0.8192 | 0.0000 | 0.0002 | 0.7503 |
| Glutarate semialdehyde/alpha-ketoisovaleric acid | 0.0222 | 0.5732 | 2.4036 | 0.8114 | -0.3016 | 0.0003 | 0.0034 | 0.7077 |
| Niacinamide (VB3) | 0.0160 | 0.1545 | 4.2737 | 1.3835 | 0.4683 | 0.0000 | 0.0002 | 0.7636 |
| Betaine | -0.0003 | -0.0027 | 3.6598 | 1.1136 | 0.1553 | 0.0343 | 0.0973 | 0.6224 |
| L-laline | -0.0003 | -0.0027 | 3.6598 | 1.1136 | 0.1553 | 0.0343 | 0.0973 | 0.6224 |
| L-lactic acid/TF-methoxyacetic acid/glyceraldehyde | -0.0025 | -0.0647 | 3.8911 | 0.6586 | -0.6025 | 0.0008 | 0.0061 | 0.6941 |
| D-glutamic acid | -0.0045 | -0.2459 | 1.7871 | 1.1821 | 0.2413 | 0.0033 | 0.0187 | 0.6700 |
| Gluconic acid | 0.0006 | 0.0382 | 2.0524 | 2.0828 | 1.0585 | 0.0000 | 0.0005 | 0.7398 |
| Hypoxanthine | 0.0191 | 0.3529 | 2.2698 | 2.0168 | 1.0121 | 0.0000 | 0.0000 | 0.7669 |
| Creatine | -0.0032 | -0.0550 | 4.0528 | 1.1988 | 0.2616 | 0.0000 | 0.0002 | 0.7540 |
| Citrulline | -0.0013 | -0.1493 | 1.0043 | 1.1938 | 0.2556 | 0.0128 | 0.0487 | 0.6439 |
| Glycerophosphocholine | -0.0202 | -0.2281 | 3.2868 | 1.3146 | 0.3946 | 0.0144 | 0.0528 | 0.6415 |
| Malic acid | 0.0210 | 0.4493 | 1.6534 | 1.2688 | 0.3434 | 0.0002 | 0.0018 | 0.7179 |
| Creatinine | 0.0053 | 0.2344 | 9.5570 | 0.5657 | -0.8219 | 0.0000 | 0.0000 | 0.7867 |
| (S)-2-methylmalate/(R)-2-methylmalate/2-hydroxyglutarate | -0.0013 | -0.0572 | 1.1719 | 1.4839 | 0.5694 | 0.0000 | 0.0000 | 0.7652 |
| 3-Methyl-2-oxovaleric acid/2-ketohexanoic acid | 0.0256 | 0.3965 | 8.7187 | 0.7488 | -0.4173 | 0.0000 | 0.0000 | 0.7917 |
| Acetylcholine/deoxycarnitine | 0.0037 | 0.0363 | 1.1108 | 1.1938 | 0.2555 | 0.0111 | 0.0439 | 0.6468 |
| N6,N6,N6-trimethyl-L-lysine | -0.0006 | -0.0033 | 1.0936 | 1.3248 | 0.4058 | 0.0021 | 0.0129 | 0.6779 |
| Ergothioneine | 0.1069 | 0.4718 | 1.2746 | 1.3426 | 0.4250 | 0.0498 | 0.1291 | 0.6134 |
| Methacholine-like | 0.0027 | 0.0825 | 1.2728 | 0.5801 | -0.7855 | 0.0052 | 0.0255 | 0.6614 |
| N-acetyl-DL-serine | -0.0091 | -0.1332 | 1.9828 | 1.2671 | 0.3416 | 0.0000 | 0.0005 | 0.7431 |
| Tagatose/gulose/fructose/galactose/mannose/sorbose/allose | 0.0046 | 0.5364 | 1.7653 | 0.8827 | -0.1799 | 0.0018 | 0.0117 | 0.6806 |

S-plot p: the magnitude variable of the S-plot X dataset; S-plot p(corr): the reliability value of the S-plot X dataset; VIP: the variable importance in projection score; FC: fold change; log_2_(FC): log_2_ value of fold change; P value: P value of the Wilcoxon signed-rank test; FDR: false discovery rate; AUC: area under the ROC curve.

**Table S3. ROC curve analysis in atrial appendage samples and plasma samples of AF and non-AF patients.**

| Metabolite | ROC curve analysis in atrial appendage samples | | | | |  | ROC curve analysis in plasma samples | | | | |
| --- | --- | --- | --- | --- | --- | --- | --- | --- | --- | --- | --- |
|  | AUC | 95% CI | P value | Sensitivity (%) | Specificity (%) |  | AUC | 95% CI | P value | Sensitivity (%) | Specificity (%) |
| A | 1.000 | 1.000-1.000 | <0.001 | 1.000 | 1.000 |  | 0.670 | 0.558-0.782 | 0.003 | 0.869 | 0.556 |
| B | 1.000 | 1.000-1.000 | <0.001 | 1.000 | 1.000 |  | 0.787 | 0.692-0.881 | <0.001 | 0.655 | 0.833 |
| C | 0.883 | 0.785-0.980 | <0.001 | 0.933 | 0.760 |  | 0.724 | 0.621-0.827 | <0.001 | 0.667 | 0.722 |
| D | 0.933 | 0.864-1.000 | <0.001 | 0.933 | 0.880 |  | 0.764 | 0.677-0.850 | <0.001 | 0.631 | 0.889 |
| E | 1.000 | 1.000-1.000 | <0.001 | 1.000 | 1.000 |  | 0.767 | 0.677-0.850 | <0.001 | 0.702 | 0.722 |
| AB | 1.000 | 1.000-1.000 | <0.001 | 1.000 | 1.000 |  | 0.855 | 0.778-0.932 | <0.001 | 0.738 | 0.833 |
| AC | 1.000 | 1.000-1.000 | <0.001 | 1.000 | 1.000 |  | 0.777 | 0.679-0.875 | <0.001 | 0.667 | 0.833 |
| AD | 1.000 | 1.000-1.000 | <0.001 | 1.000 | 1.000 |  | 0.797 | 0.713-0.882 | <0.001 | 0.857 | 0.639 |
| AE | 1.000 | 1.000-1.000 | <0.001 | 1.000 | 1.000 |  | 0.765 | 0.662-0.850 | <0.001 | 0.786 | 0.611 |
| ABC | 1.000 | 1.000-1.000 | <0.001 | 1.000 | 1.000 |  | 0.927 | 0.875-0.979 | <0.001 | 0.905 | 0.833 |
| ABD | 1.000 | 1.000-1.000 | <0.001 | 1.000 | 1.000 |  | 0.892 | 0.829-0.954 | <0.001 | 0.929 | 0.722 |
| ABE | 1.000 | 1.000-1.000 | <0.001 | 1.000 | 1.000 |  | 0.874 | 0.802-0.946 | <0.001 | 0.786 | 0.833 |
| ACD | 1.000 | 1.000-1.000 | <0.001 | 1.000 | 1.000 |  | 0.827 | 0.748-0.906 | <0.001 | 0.762 | 0.750 |
| ACE | 1.000 | 1.000-1.000 | <0.001 | 1.000 | 1.000 |  | 0.816 | 0.732-0.900 | <0.001 | 0.702 | 0.861 |
| ADE | 1.000 | 1.000-1.000 | <0.001 | 1.000 | 1.000 |  | 0.831 | 0.753-0.909 | <0.001 | 0.810 | 0.750 |

The metabolites A, B, C, D, and E in the first column represent D-glutamic acid, creatinine, choline, niacinamide, and hypoxanthine, respectively. AUC: area under curve; 95% CI: 95% Confidence interval; P value: null hypothesis of the true area; Cutoff value: the maximum of Youden index.


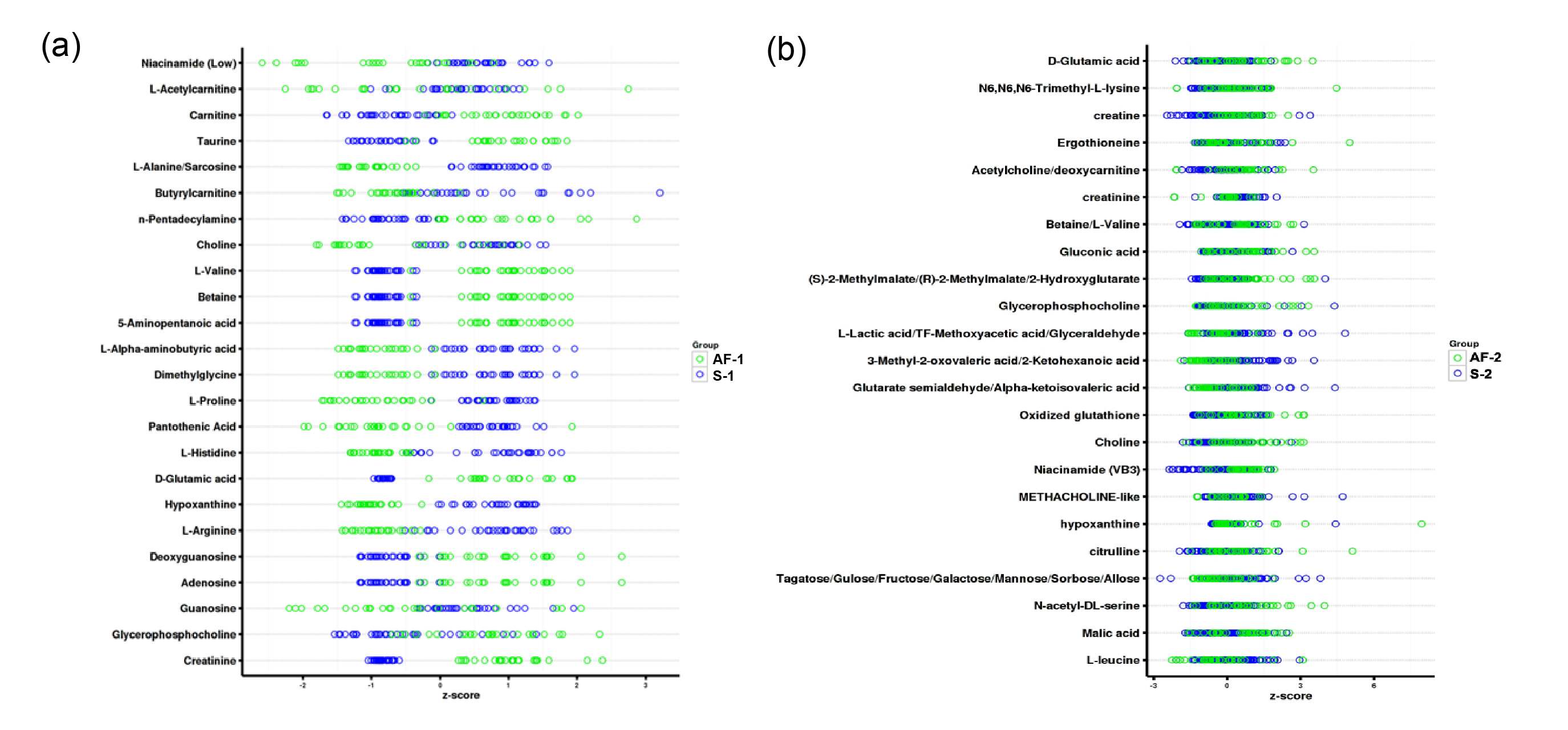


**Figure S1. Z-score of differential metabolites.** (**a**) Z-score of differential metabolites about atrial appendage samples. Scores focus in the range of -2 to 3. (**b**) Z-score of differential metabolites about plasma samples. Scores focus in the range of -3 to 4. AF-1 and AF-2 are for atrial appendage and plasma of AF cases group; S-1 and S-2 are for atrial appendage and plasma of non-AF group.
